# Blisters to Prevent Abortion

**Published:** 1828-10

**Authors:** 


16. *Blisters to prevent abortion.*—In the August number (1828) of the *American Journal of the Medical and Physical Sciences*, Dr. Samuel Jackson, of Northumberland, Pa. well known to the profession in the United States, as the author of several valuable papers, has inserted a communication on the efficacy of blisters in preventing Abortion. He does not, as yet, venture to insist upon them, as a remedy for the miscarriages which occur to the same patients, many times in succession,—and which constitute one of the *opprobria medicorum*,—but as a promising acquisition to the means already in use, for preventing abortion from occasional or accidental causes; and that cases of this kind, too often call for other aid than that which is generally employed, every physician of extensive practice must admit. That Dr. J. may speak to our readers for himself, we shall extract the following interesting case:

"*Case 2.*—About two months since, we were sent for to visit the wife of P. L. in Sunbury, but not being able to attend for some time, we found, on arriving at the house, that our friend, Dr. Price of that town had been called. The patient, in the sixth month of her pregnancy, had made a misstep the day before, from which she suffered some pain in her back during the whole night; in the morning she walked out, but found herself worse for it, and, upon returning home, she was seized with bearing pains, followed by such a discharge of water as led her to suppose that the membranes were ruptured: the pain increased, with frequent gushes of water, and by the time Dr. Price had arrived, it was excessively violent, coming on at regular intervals of about five minutes and reaching down the thighs; she had also a severe strangury which obliged her to rise frequently and pass a few drops of urine with great pain. Dr. P. had directed an enema of laudanum, half an ounce, which she had retained without any alleviation, though at least an hour and a half had now elapsed.

The *os uteri* was, as we believed, a very little patulous, there had been some sanguinous show, and after the most careful inquisition, we presumed that the membranes had given way, and the water passed off. The uterus was in its proper site, the pains did not seem to affect it very considerably, and therefore even if abortion were to be considered as finally inevitable, these were not the proper pains to produce it, and hence they were to be subdued if possible. Her pulse was too feeble to admit of bleeding, and moreover she was of a peculiar constitution, and seldom profited by the loss of blood. Solid opium was now given in doses of five grains, with the hope that in conjunction with the enema already taken, it would subdue these pains and prepare the system for the supervention of genuine labour. But these potent doses produced very partial, and only temporary relief, and did not fail to bring on severe vomiting, which always renewed the pressing pains. Two indications therefore arose—to subdue the pains, and to compose the stomach. The black drop and paregoric elixir she abhorred, and hence we continued to give solid opium by the stomach, and laudanum by injection; but no sooner did the pain abate under these anodynes, than a dreadful vomiting ensued, which constantly renewed it. We endeavoured to keep the system under the steady influence of opiates, being careful to administer some as soon as the effects of the preceding dose began to subside into languor and sickness; we tried to compose the stomach with ether, elixir vitriol, lupuline, ginger tea, an article we have found of great service in the nausea left by opium. But all these applications were vain; either vomiting or pain, and sometimes both, were continually present, and the state of the poor sufferer was truly alarming. We must confess that in such a case the acetate of morphia, or the denarcotised laudanum ought to have been used, but we had rather unjustifiably neglected to procure these articles, having always found an acetic preparation on the principles of the *black drop*, to answer as a succedaneum to common laudanum.

Here let us observe that we should have applied a blister to the lumbar region at first, had it not been that we looked upon abortion as inevitable, and considered that genuine labour would soon supervene. But now, after a struggle of two days and nights, without any advantage being gained, we proposed to our colleague the application of a blister, and he readily consented, though he acknowledged the practice to be entirely new to him. Just in proportion as the plaster drew, the pain receded, and was felt no more after the blister was fully drawn. So complete was the cure that a tremendous fire the following night, which suddenly consumed a large wooden barn within twenty perches of her house, and alarmed her excessively, did not renew the pains. Even the strangury was entirely removed by the blister, and did not return. She has continued well ever since, and she now feels the fœtus as vigorous as before. She was of opinion that at least half a gallon of water was gradually discharged in the course of the first two days. That the liquor amnii may pass off, and the fœtus survive, is probably incontrovertible, but it certainly occurs so seldom as to be justly considered a very rare exception to the general course of things. That it occurred in this case we cannot by any means aver; the lady may have been deceived in the quantity passed, and the water may have come from one of those collections which authors describe as sometimes existing between the membranes."

In a subsequent part of his paper Dr. J. has the following general remarks on the safety of blistering in the pregnant state, to which we are disposed to extend an entire concurrence.

"It has been said that blisters are to be avoided as far as possible in all cases of pregnancy, because they are particularly apt to produce strangury in this state of the body, an affection that might be attended with dangerous consequences. But we cannot forbear asserting, that we have never hesitated to apply them on account of pregnancy, and that we do not recollect having ever seen a strangury from them under such circumstances. This exemption may be owing to the care we have very generally had of giving parsley tea or some diuretic during the whole time of their drawing. Last summer a lady in the ninth month of her pregnancy, suffered severely with œdematous legs, and having heard of blisters in such cases, she proposed the remedy herself; we tried to dissuade her from using them, but she did it clandestinely, and had great cause to rejoice in her temerity."

We shall conclude this notice with a reminiscence which cannot be regarded as foreign to it.

The nausea and vomiting attendant on pregnancy, are well known to be sometimes most obstinate and distressing. They may even eventuate in abortion. Some years since, a patient fell under our observation, who throughout the whole period of two pregnancies, had experienced distressing and almost uninterrupted nausea, with great consequent reduction of strength and flesh. She was then in her third term, and affected with the same disturbance of the stomach: In this afflicting situation, a pair of blisters were applied to the ankles, and gave immediate and permanent relief.

